# Markers of Thrombogenesis and Fibrinolysis and Their Relation to Inflammation and Endothelial Activation in Patients with Idiopathic Pulmonary Arterial Hypertension

**DOI:** 10.1371/journal.pone.0082628

**Published:** 2013-12-02

**Authors:** Grzegorz Kopeć, Deddo Moertl, Sabine Steiner, Ewa Stępień, Tomasz Mikołajczyk, Jakub Podolec, Marcin Waligóra, Jakub Stępniewski, Lidia Tomkiewicz-Pająk, Tomasz Guzik, Piotr Podolec

**Affiliations:** 1 Department of Cardiac and Vascular Diseases of the John Paul II Hospital in Krakow and the Jagiellonian University Collegium Medicum, Krakow, Poland; 2 Department of Internal Medicine III (Cardiology and Emergency Medicine), Landesklinikum St. Poelten, St. Poelten, Austria; 3 Division of Angiology, Department of Internal Medicine II, Medical University of Vienna, Vienna, Austria; 4 Department of Clinical Biochemistry, Jagiellonian University Collegium Medicum, Krakow, Poland; 5 Department of Internal and Agricultural Medicine, Jagiellonian University Collegium Medicum, Krakow, Poland; 6 Department of Hemodynamics and Angiocardiography of the John Paul II Hospital in Krakow and the Jagiellonian University Collegium Medicum, Krakow, Poland; Vanderbilt University Medical Center, United States of America

## Abstract

**Background:**

Chronic anticoagulation is a standard of care in idiopathic pulmonary arterial hypertension (IPAH). However, hemostatic abnormalities in this disease remain poorly understood. Therefore, we aimed to study markers of thrombogenesis and fibrinolysis in patients with IPAH.

**Methods:**

We studied 27 consecutive patients (67% female) with IPAH aged 50.0 years (IQR: 41.0 - 65.0) and 16 controls without pulmonary hypertension. Prothrombin fragment 1+2 (F1+2) and thrombin-antithrombin (TAT) complexes were measured to assess thrombogenesis; tissue-type plasminogen activator (tPA) antigen and plasmin-anti-plasmin complex to characterize activation of fibrinolysis; plasminogen activator inhibitor 1 (PAI-1) to measure inhibition of fibrinolysis; and endothelin-1 (ET-1) and interleukin-6 (IL-6) to assess endothelial activation and systemic inflammation, respectively. In addition, in treatment-naive IPAH patients these markers were assessed after 3 months of PAH-specific therapies.

**Results:**

TPA (10.1[6.8-15.8] vs 5.2[3.3-7.3] ng/ml, p<0.001), plasmin-anti-plasmin (91.5[60.3-94.2] vs 55.8[51.1-64.9] ng/ml, p<0.001), IL-6 (4.9[2.5-7.9] vs 2.1[1.3-3.8] pg/ml, p=0.001) and ET-1 (3.7 [3.3-4.5] vs 3.4[3.1-3.5], p= 0.03) were higher in patients with IPAH than in controls. In IPAH patients plasmin-anti-plasmin and tPA correlated positively with IL-6 (r=0.39, p=0.04 and r=0.63, p<0.001, respectively) and ET-1 (r=0.55, p=0.003 and r=0.59, p=0.001, respectively). No correlation was found between tPA or plasmin-anti-plasmin and markers of thrombogenesis. Plasmin-anti-plasmin decreased after 3 months of PAH specific therapy while the other markers remained unchanged.

**Conclusions:**

In the present study we showed that markers of fibrynolysis were elevated in patients with IPAH however we did not find a clear evidence for increased thrombogenesis in this group of patients. Fibrinolysis, inflammation, and endothelial activation were closely interrelated in IPAH.

## Introduction

Histopathological studies have shown a high prevalence of in situ thrombosis in patients with idiopathic pulmonary arterial hypertension (IPAH) [1,2]. Additionally, some observational studies suggested a survival benefit in patients with IPAH when taking oral anticoagulation [3]. Therefore, the use of vitamin K antagonists (VKA) has become a standard of care in IPAH [4]. 

 This indication, however, has been questioned recently with the introduction of advanced PAH specific therapy which relieved many patients from bed resting [5,6]. Additionally, recent data suggested an increased bleeding risk in patients with IPAH compared to patients taking VKA for other reasons [7].

 Generally, increased thrombotic or bleeding risk might stem from an altered balance between procoagulant, anticoagulant and fibrinolytic activity. The thrombin mediated enzymatic conversion of fibrinogen to fibrin is the major step in clot formation. Typically, in response to the generation of fibrin thrombus in vivo there is an activation of the fibrinolytic system [8]. Currently, the characteristics of these two counteracting processes in IPAH are poorly known.

 Therefore, we aimed to characterize the process of thrombogenesis and fibrinolysis activation in patients with IPAH. As thrombogenesis and fibrinolysis in systemic circulation are interrelated with inflammation and endothelial function we also measured markers of inflammation and endothelial activation.

## Methods

### Study population

**Figure 1:**
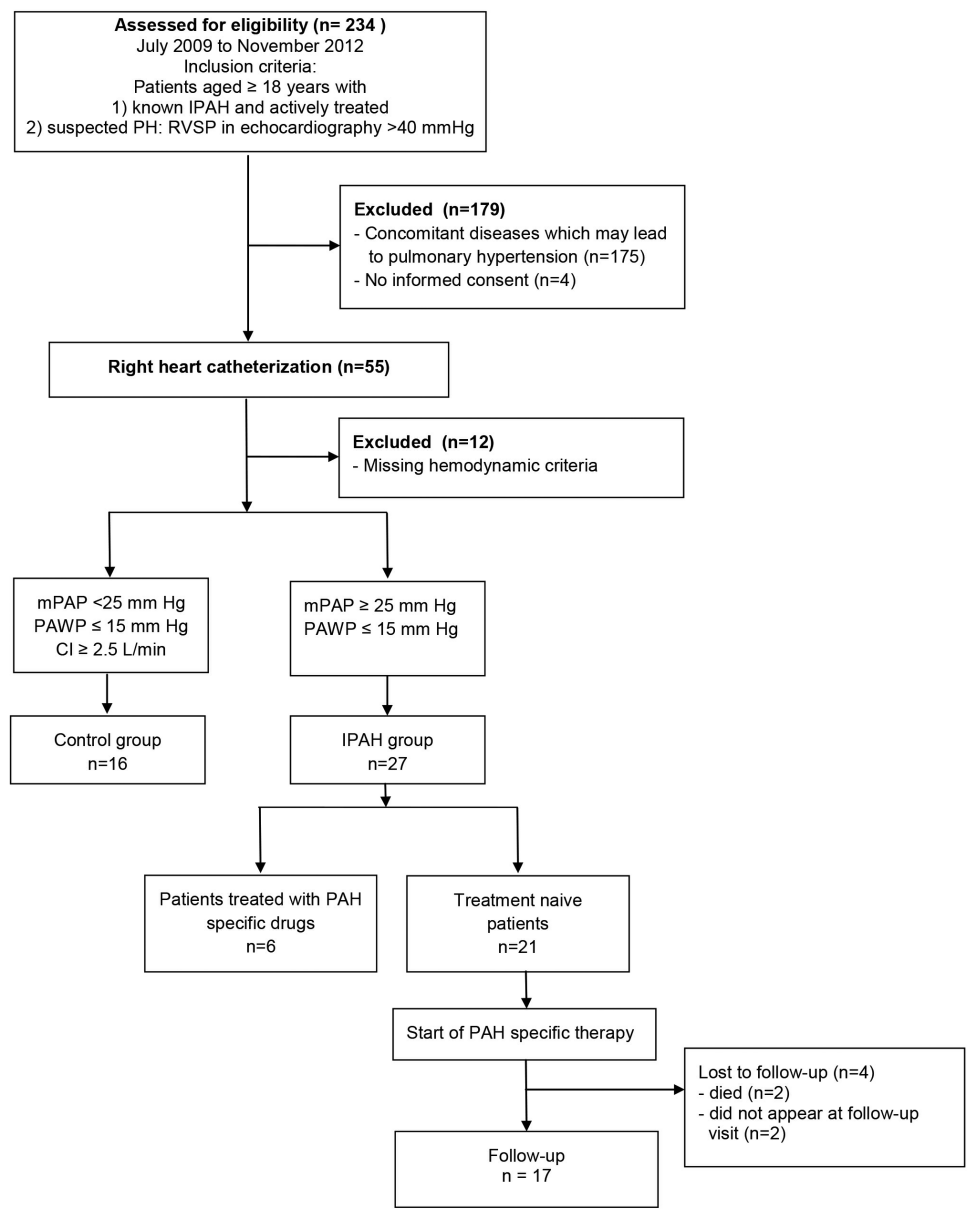
Study flow chart explaining selection of participants of the study and depicting the follow-up scenario. CI, cardiac index; IPAH, idiopathic pulmonary arterial hypertension; mPAP, mean pulmonary artery pressure; PAWP, pulmonary artery wedge pressure.

 All study participants were recruited consecutively at the Department of Cardiac and Vascular Diseases at John Paul II Hospital in Krakow, Poland between July 2009 and November 2012 as shown in Figure 1. 

 Eligible patients were 1) patients with known IPAH scheduled for routine follow-up visit and 2) patients with suspected IPAH referred for diagnosis and management at our pulmonary hypertension (PH) centre. The latter group consisted of all patients, irrespectively of symptoms, in whom echocardiography showed increased right ventricular systolic pressure >40 mmHg [9] and in whom right heart catheterization was made in order to confirm or exclude the diagnosis of pulmonary hypertension. To be included in the study group secondary aetiologies of PH (liver disease, HIV infection, connective tissue disease, congenital heart defect, left heart disease, chronic thromboembolic disease and lung diseases) had to be excluded. Additional exclusion criteria were age ≤ 18 years, acute infection or apparent inflammatory process. Patients with mean pulmonary artery pressure (mPAP) ≥25mmHg and pulmonary artery wedge pressure (PAWP) ≤15 mmHg were diagnosed as IPAH. Patients with mPAP < 25 mmHg, PAWP ≤15mmHg, and a cardiac index (CI) ≥ 2.5 L/min/m^2^ were taken as controls. We considered the result of echocardiography (RVSP >40 mmHg) in the latter group as false positive. The institutional ethics committee (Komisja Bioetyczna przy Okręgowej Izbie Lekarskiej w Krakowie) approved the study protocol, and written informed consent was obtained from each patient before starting the study. All clinical investigations have been conducted according to the principles expressed in the Declaration of Helsinki.

### Study protocol

 Data on medical history, physical examination and laboratory parameters were obtained before cardiac catheterization. Patients with newly diagnosed IPAH were invited for a follow-up examination 3 months after initiation of PAH specific therapy. Cardiovascular risk factors were assessed according to current guidelines[10-14]. A smoker was defined as having smoked at least for one month during the previous 12 months; all others were classified as non-smokers. Body mass index was calculated as mass[kg]/height[m]^2^. Coronary angiography was performed in each patient to screen for coronary artery disease.

### Cardiac catheterisation

 Right heart catheterization (RHC) was performed in supine position from the right femoral vein access using a Swan-Ganz catheter in every patient. The acquisition of pressure waves was made at end expiration. Cardiac output was measured using the Fick oxygen consumption method. Blood oxygen saturation was measured with Co-oximeter OSM3 (Radiometer, Copenhagen, Denmark) while the oxygen consumption was estimated from the LaFarge equation as previously described [15]. Pulmonary vascular resistance was calculated as the difference between mPAP and PAWP divided by cardiac output. 

### Measurement of laboratory parameters

 The venous blood samples were taken from each patient after an overnight fast in the morning on the day of catheterization. Additionally, in patients with newly diagnosed IPAH, repeat venous blood samples were taken three months after initiation of PAH specific therapy. Patients who were previously treated with VKA (only patients with prior diagnosis of IPAH) were switched to enoxaparine at least three days before RHC and had to have an INR < 1.2 when the blood was taken for further analysis. Patients did not receive enoxaparine in the morning of the day when blood samples were taken for analysis. 

 Blood samples were taken from the antecubital vein using sodium citrate (0,109 M) or EDTA as anti-coagulants as recommended by the manufacturer’s instructions for the assays and centrifuged within one hour after collection (2500 g, 20 min). Following the current recommendations [16] the second tube was used for the coagulation specimen. Plasma samples were stored in aliquots at −80°C. Immunoassays were performed according to manufacturers’ instructions.

 Prothrombin fragment 1+2 (F1+2) and thrombin-antithrombin (TAT) complexes were measured to assess thrombogenesis. Tissue-type plasminogen activator (tPA) antigen and plasmin-anti-plasmin complex were measured to characterize activation of fibrinolysis. D-Dimer was chosen to reflect both processes. Plasminogen activator inhibitor 1 (PAI-1) was measured to assess inhibition of fibrinolysis. Endothelin-1 (ET-1) was used as a marker of endothelial activation, and interleukin-6 (IL-6) as a marker of chronic systemic inflammation.

 F1+2 was assessed in citrate plasma with ELISA kit Human Prothrombin Fragment 1+2 (F1+2) (USCN Life Science Inc, TX, USA). TAT complexes concentrations were determined in citrate plasma by means of ELISA assay IMUBIND TAT ELISA (American diagnostica GmbH, Pfungstadt, Germany). TPA antigen levels were measured in citrate plasma by means of a one-step two-site immuno-assay Zymutest tPA antigen (BioMed, Neuville-sur-Oise, France). Plasmin-anti-plasmin complex concentrations were determined in citrate plasma by means of ELISA assay Plasmin-anti-plasmin Assay Kit (USCN Life Science Inc; Wuang, China). D-dimer levels were measured in citrate plasma by means of ELISA Zymutest D-dimer (Hyphen BioMed, Neuville-sur-Oise, France). PAI-1 levels were measured in citrate plasma by means of a one-step two-site immuno-assay Zymutest PAI-1 activity (Hyphen BioMed, Neuville-sur-Oise, France). The concentration of ET-1 was assessed in EDTA plasma by means of ELISA assay Human Endothelin-1 Quantikine (R&D Systems, MN, USA). The concentration of IL-6 was assessed in EDTA plasma by means of ELISA assay Human IL-6 Quantikine HS (R&D Systems, MN, USA). 

### Statistical analysis

 Continuous variables were reported using median and interquartile range, categorical variables were described as counts and percentages. Patients with IPAH and controls were compared with the Mann Whitney U-test for the continuous variables and with the chi square test for categorical variables. The Wilcoxon rank sum test was used to compare parameters at baseline and after three months of treatment. Spearman rank correlation was used to assess the association between continuous variables. The significance level was set at p < 0.05. Statistical analysis was performed with Statistica PL software [StatSoft, Inc. (2010). STATISTICA (data analysis software system), version 9.1. Tulsa, USA www.statsoft.com] and MedCalc version 11.6.1.0 (MedCalc Software, Mariakerke, Belgium).

## Results

### Baseline characteristics

 Among 234 patients who were assessed for eligibility (Figure 1), 191 patients were excluded due to other aetiologies of pulmonary hypertension (n=175), lack of informed consent (n=4), and missing hemodynamic criteria (n=12). The study population consisted of 43 participants: 27 patients with IPAH and 16 controls.

 No differences in age and sex were found between patients and controls. At baseline, the majority of patients (n = 21; 78%) were treatment naive; the others were treated with sildenafil (2 patients), sildenafil and inhaled iloprost (1 patient), inhaled iloprost (1 patient), treprostinil s.c. with sildenafil (1 patient), and sitaxsentan (1 patient). After confirming the diagnosis of IPAH all drug-naive patients received PAH specific drugs: Fifteen patients were prescribed sildenafil, 2 verapamil, 1 inhaled iloprost, 1 sildenafil + inhaled iloprost and 2 subcutaneous treprostinil. Patient characteristics involving cardiovascular risk factors, cardiovascular drugs used before study enrolment and hemodynamic data are presented in Table 1. Seventeen (63%) IPAH patients were in WHO functional class III, 8 patients in functional class II and 2 patients in class IV at study entry. All but one IPAH patients and all controls had normal coronary arteries. 

**Table 1:** Patient characteristics.

Variable	IPAH (n=27)	Controls (n=16)	P
Age [years]	50.0 (41.0-65.0)	54.0 (37.0-60.5)	0.76
Sex [males]	9 (33%)	5 (31%)	0.84
BMI [kg/m^2^]	23.0 (21.0-26.0)	27.3 (22.3-28.3)	0.02
Total cholesterol [mmol/l]	4.1 (3.6-4.7)	4.7 (4.6-5.3)	0.007
LDL-cholesterol [mmol/l]	2.4 (1.7-2.8)	2.7 (2.5-3.5)	0.03
HDL-cholesterol [mmol/l]	1.3 (1.1-1.5)	1.6 (1.3-1.8)	0.01
Triglycerides [mmol/l]	1.0 (0.8-1.4)	1.1 (0.9-1.4)	0.7
Diabetes	4 (15%)	0	0.28
Systemic arterial hypertension	10 (37%)	8 (50%)	0.61
Smoking	0	0	-
hsCRP [mg/dl]	2.4 (1.2-8.2)	1.1 (0.7-2.2)	0.004
Creatinine [umol/l]	80.0 (73.0-98.0)	68.5 (59.0-88.5)	0.02
Drugs taken before entering the study			
Statins	9 (33%)	6 (38%)	0.96
Diuretics	12 (44%)	0	0.005
ACEI	6 (22%)	6 (38%)	0.47
Beta blockers	8 (30%)	6 (38%)	0.80
CCB	8 (30%)	2 (12.5%)	0.36
Vitamin K antagonist*	6	0	-
Hemodynamic data			
PA mean pressure [mmHg]	54.0 (44.3-66.3)	11.0 (9.5-13.0)	<0.001
RA pressure [mmHg]	8.0 (5.0-13.0)	2.0 (2.0-3.0)	<0.001
PAW mean pressure [mmHg]	8.0 (4.5-11.3)	7.5 (5.5-9.0)	0.36
Ao mean pressure [mmHg]	90.0 (79.0-96.8)	100.0 (94.0-102.0)	0.02
Cardiac index [l/min/m^2^]	1.6 (1.4-1.9)	2.7 (2.7-2.8)	<0.001
Estimated oxygen consumption (ml/min)	187.5 (176.2-203.1)	180.6 ( 160.9 - 270.6)	0.58
PVR [WU]	15.9 (11.3-20.9)	0.7 (0.4-1.3)	<0.001

* only patients with known IPAH were treated with vitamin K antagonists before enrolment to the study

ACEI - angiotensin converting enzyme inhibitor, BMI - body mass index, CCB - calcium channel blockers, HDL - high density lipoprotein, LDL - low density lipoprotein, PA - pulmonary artery, PAW - pulmonary artery wedge, PVR - pulmonary vascular resistance, RA - right atrium, WU - Wood Units

 Twenty-one patients who were treatment naive at recruitment received PAH specific drug treatment after baseline measurements and were followed for 3 months. In 17 of these 21 patients we were able to reassess the laboratory markers after 3 months. From the remaining 4 patients 2 patients died during the follow up period, and 2 patients did not appear at the 3-months follow-up visit (Figure 1).

### Markers of haemostasis, inflammation and endothelial dysfunction

**Figure 2:**
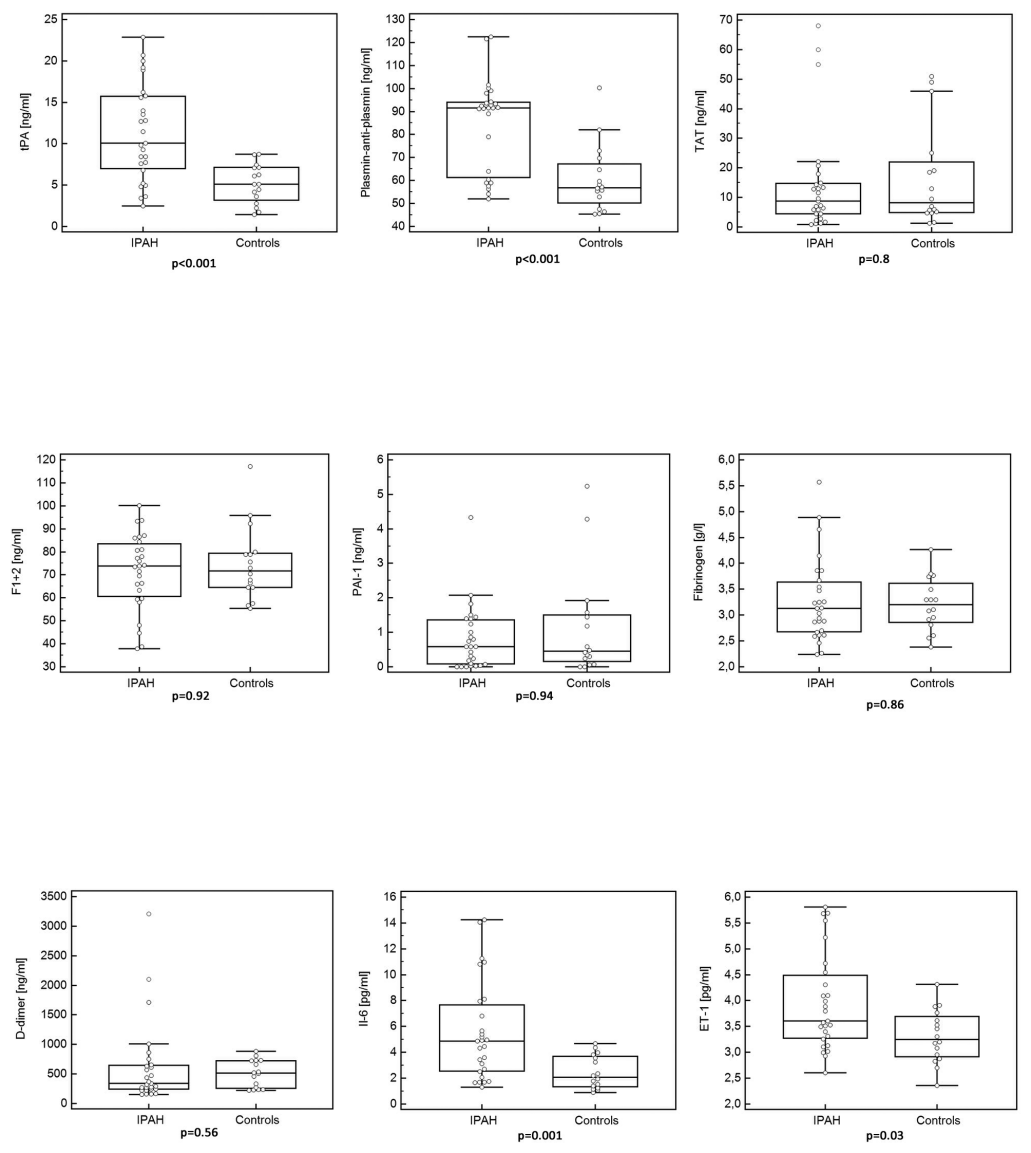
Comparison of hemostatic markers, Interleukin-6, and Endothelin-1 between patients with idiopathic arterial hypertension and controls. ET-1 - endothelin 1, F1+2 - prothrombin fragments 1+2, IL-6 - Interleukin 6, PAI-1 - plasminogen activator inhibitor 1, TAT - thrombin-antithrombin, tPA - tissue plasminogen activator. In this box-and-whisker plot, the central box represents the values from the lower to upper quartile (25 to 75 percentile). The middle line represents the median. A line extends from the minimum to the maximum value, excluding values that are smaller than the lower quartile minus 1.5 times the interquartile range, or larger than the upper quartile plus 1.5 times the interquartile range.

 Both markers of fibrinolysis, tPA and plasmin-anti-plasmin, were significantly higher in patients with IPAH than in controls (Table 2, Figure 2). In contrast, markers of thrombogenesis, TAT and F1+2, as well as PAI-1 and D-dimer did not differ between patients with IPAH and controls. IL-6 and ET-1 were higher in IPAH patients than in controls (Table 2, Figure 2). Similar results were obtained when only treatment naive IPAH patients were included in the analysis.

**Table 2:** Markers of hemostasis, Interleukin-6 and Endothelin-1 in patients with IPAH and controls.

Parameter	IPAH (n=27)	Controls (n=16)	P
tPA [ng/ml]	10.1 (6.8-15.8)	5.2 (3.3-7.3)	<0.001
Plasmin-anti-plasmin [ng/ml]	91.5 (60.3-94.2)	55.8 (51.1-64.9)	<0.001
TAT (ng/ml)	8.7 (4.5-14.9)	8.2 (4.9-22.2)	0.8
F1+2 (ng/ml)	73.9 (59.7-84.4)	71.8 (63.5-78.9)	0.92
PAI-1 (ng/ml)	0.58 (0.1-1.4)	0.46 (0.2-1.5)	0.94
Fibrinogen (g/l)	3.1 (2.7-3.7)	3.2 (2.8-3.5)	0.86
D-dimer (ng/ml)	342.8 (243.1-648.9)	513.4 (263.5-732.7)	0.56
Il-6 (pg/ml)	4.9 (2.5-7.9)	2.1 (1.3-3.8)	0.001
ET-1 (pg/ml)	3.7 (3.3-4.5)	3.4 (3.1-3.5)	0.03

ET-1 - endothelin-1, F1+2 - prothrombin fragments 1+2, IL-6 - interleukin 6, tPA- tissue plasminogen activator, PAI-1 - plasminogen activator inhibitor, TAT - thrombin-anti-thrombin

 We did not observe a significant effect of previous treatment with VKA on the markers of thrombogenesis. F1+2 (76.0 [60.2-79.8] vs 73.7 [61.5-85.2], p =0.87) and TAT (8.7 [5.8-13.3] vs 8.4 [3.6-16.4], p=0.82) were similar in IPAH patients previously treated with VKA and those without prior VKA intake. 

**Figure 3:**
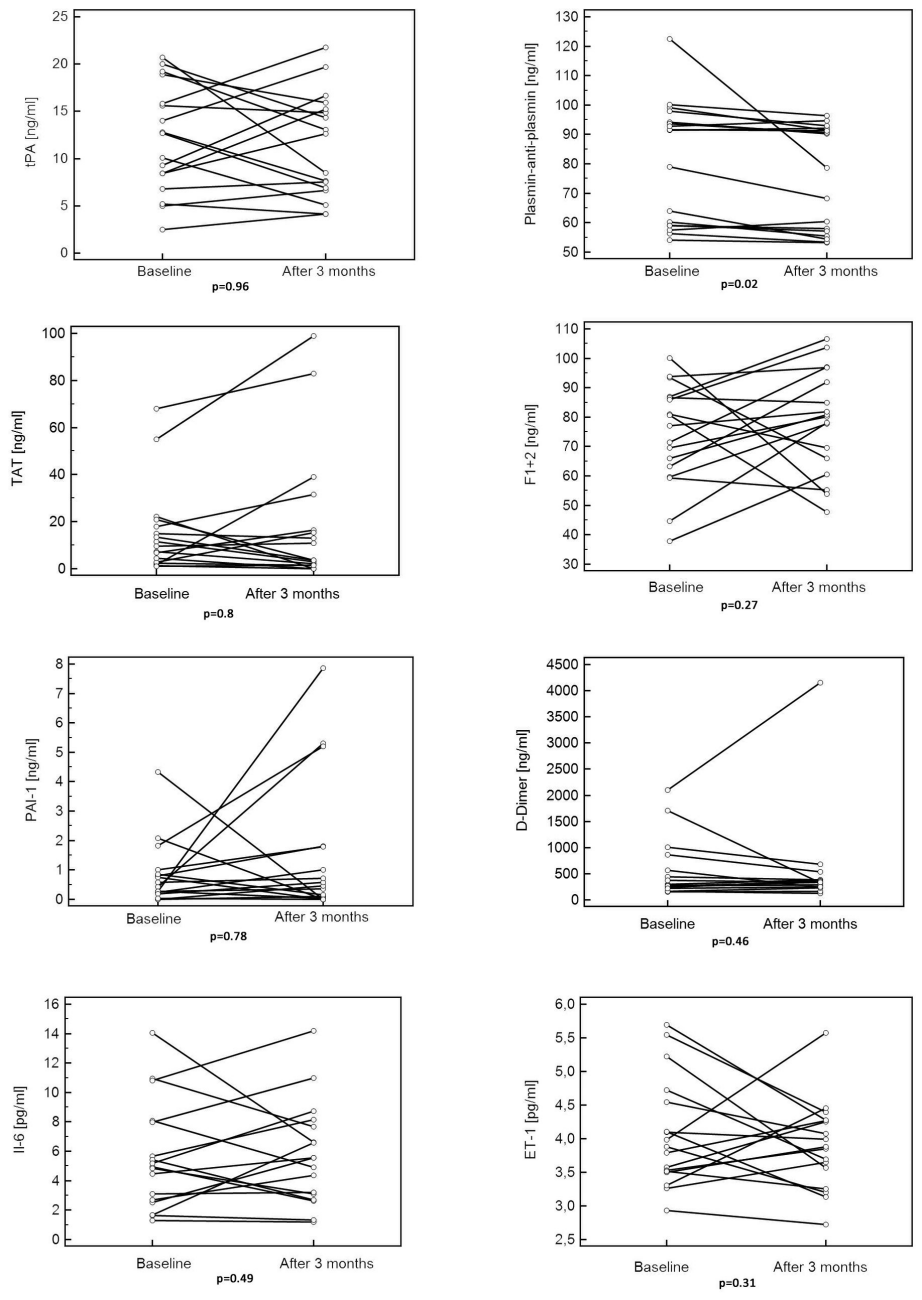
Levels of haemostatic variables, Interleukin-6 and Endothelin-1 at baseline and after 3 months of pulmonary arterial hypertension specific therapy. ET-1 - endothelin-1, F1+2 - prothrombin fragments 1+2, IL-6 - interleukin 6, PAI-1 - plasminogen activator inhibitor, TAT - thrombin-anti-thrombin, tPA- tissue plasminogen activator.

 In previously drug-naive IPAH patients we observed a reduction of plasmin-anti-plasmin levels after 3 months of treatment with PAH specific drugs, while tPA, D-Dimer, TAT, F1+2, PAI-1, and IL-6, ET-1 remained unchanged (Table 3, Figure 3).

**Table 3:** Median change of markers of hemostasis, Interleukin-6, and Endothelin-1 after 3 months of PAH specific treatment in patients who were treatment naive at study entry.

Parameter	Median (IQR)	P
tPA [ng/ml]	0.01 (-5.1 - 4.9)	0.96
Plasmin-anti-plasmin [ng/ml]	-2.9 (-5.6 - -0.2)	0.02
TAT (ng/ml)	-0.7 (-5.9 - 12.9)	0.8
F1+2 (ng/ml)	9.0 (-4.2 - 19.5)	0.27
PAI-1 (ng/ml)	0.09 (-0.9 - 0.5)	0.78
D-Dimer (ng/ml)	6.2 (-326.4 - 67.1)	0.46
Il-6 (pg/ml)	0.6 (-2.3 - 3.0)	0.49
ET-1 (pg/ml)	-0.2 (-1.0 -0.4)	0.31

ET-1 - endothelin-1, F1+2 - prothrombin fragments 1+2, IL-6 - interleukin 6, IQR - interquartile range, tPA- tissue plasminogen activator, PAI-1 - plasminogen activator inhibitor, TAT - thrombin-anti-thrombin

### Correlations of tPA and plasmin-anti-plasmin in patients with IPAH

**Figure 4:**
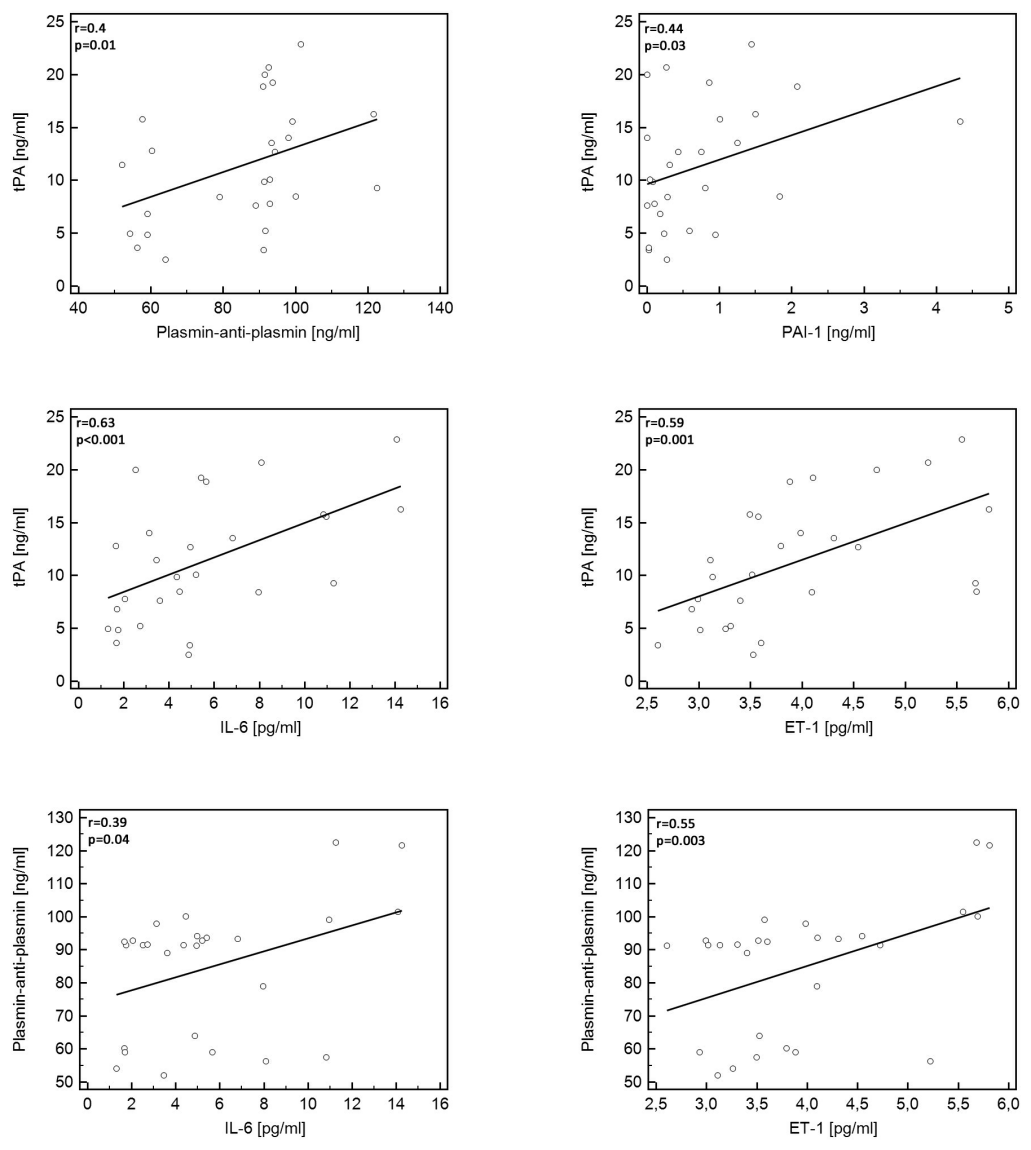
Significant correlations between hemostatic markers, Endothelin-1, and Interleukin-6. ET-1 - Endothelin -1, IL-6 - Interleukin 6, PAI-1 - PAI-1 - plasminogen activator inhibitor 1, tPA - tissue plasminogen activator.

 A positive correlation was found between tPA and plasmin-anti-plasmin and between tPA and PAI-1 (Table 4, Figure 4) in patients with IPAH. Furthermore, both plasmin-anti-plasmin and tPA correlated positively with IL-6 and ET-1, respectively (Table 4, Figure 4). No correlation was found between tPA or plasmin-anti-plasmin and markers of thrombogenesis (F1+2, TAT) while a non-significant trend for a positive correlation between tPA and F1+2 was observed. The analysis made after exclusion of the already treated IPAH patients confirmed the observations made on the whole study group. In the control group we found a positive correlation between tPA and PAI-1 (r=0.59, p=0.01), and fibrinogen (r=0.6, p=0.02), and between plasmin-anti-plasmin and fibrinogen (r=0.56, p=0.02).

**Table 4:** Correlations between tPA and plasmin-anti-plasmin and other haemostatic variables, Endothelin-1 and Interleukin-6 in patients with IPAH.

	tPA		Plasmin-anti-plasmin	
	r	P	r	P
				
Plasmin-anti-plasmin [ng/ml]	0.4	0.01	-	-
TAT (ng/ml)	0.1	0.64	0.03	0.86
F1+2 (ng/ml)	-0.38	0.06	-0.3	0.12
PAI-1 (ng/ml)	0.44	0.03	0.29	0.13
D-Dimer (ng/ml)	0.34	0.09	0.02	0.93
Fibrinogen	-0.01	0.68	-0.1	0.64
Il-6	0.63	<0.001	0.39	0.04
ET-1	0.59	0.001	0.55	0.003

ET-1 - endothelin-1, F1+2 - prothrombin fragments 1+2, IL-6 - interleukin 6, tPA- tissue plasminogen activator, PAI-1 - plasminogen activator inhibitor, TAT - thrombin-anti-thrombin

## Discussion

 In the present study we have shown that markers of fibrinolysis were elevated in patients with IPAH while markers of thrombogenesis were not different from controls without PH. Furthermore, we found significant correlations between markers of fibrinolysis and both inflammation as well as endothelial activation indicating that these three distinct processes are interrelated in IPAH. 

 Current data on coagulation and the fibrinolytic systems in IPAH are inconsistent. Increased thrombin activity as measured by the level of circulating fibrinopeptide A was shown in one study [17]. However thrombin activity was not different between IPAH and controls in two other studies in which F1+2 and TAT were measured [18,19]. These data were published in 1990s and have not been reproduced in the modern era of pulmonary hypertension. However the changing demographics of IPAH population [20] and diagnostic criteria for this disease [4,21] limit extrapolation of such historical data to the current patient population.

 Plasma markers of fibrinolysis such as tPA and plasmin-anti-plasmin were similar in PAH and controls in some studies [18,19] but elevated in other studies in patients with PAH associated with systemic sclerosis [8] and in women with IPAH [22]. Discrepant results were also shown for PAI-1 activity [18,19,23]. In our study, plasma levels of F1+2 and TAT complexes were similar in patients with IPAH and in controls suggesting lack of increased thrombin activation. In contrast, increased levels of tPA and plasmin-anti-plasmin complexes indicate increased fibrinolytic activity in IPAH. Activation of the fibrinolytic system – as observed in our study - does not necessarily contradict the high prevalence of thrombotic lesions found in autopsy studies of IPAH patients. A recent study has described fibrin that was resistant to lysis in patients with PH, suggesting that activated fibrinolysis might be ineffective to dissolve thrombi in the pulmonary vasculature [24]. On the other hand, activation of the fibrinolytic system could enhance the risk of bleeding. In fact, recent data from patients with PH treated with VKA demonstrated more major bleeding in IPAH and PAH associated with connective tissue disease than in CTEPH patients [7]. The authors of this study also reported a much higher rate of bleeding in this patient population than in large cohorts of patients treated with VKA for atrial fibrillation or venous thromboembolism [7].

 Apart from thrombogenesis [25,26], other pathophysiologic stimuli of fibrinolysis have been described. In particular, endothelial activation and inflammation have been linked with haemostatic abnormalities in the systemic circulation [25,27,28]. Importantly, both endothelial activation and chronic inflammation have also been shown to play a significant role [29,30] in the development of IPAH. 

 High levels of the proinflammatory cytokine IL-6 have been reported in most [31] but not all [32] studies in IPAH. The increased IL-6 production in IPAH is thought to reflect enhanced synthesis by both inflammatory and pulmonary vascular cells [33]. In our study PAH specific therapies did not change plasma levels of IL-6 which confirmed findings from previous studies on IL-6 and some other inflammatory markers [34].

In animal models [35] pre-treatment with IL-6 induced a threefold increase of a basal tPA in the systemic circulation. In observational studies in various patient populations including healthy individuals, patients at high cardiovascular risk as well as in patients with advanced liver disease [36-39] IL-6 levels have been shown to correlate with tPA, which supports IL-6 induced endothelial cell activation and subsequent release of tPA as one of the potential underlying pathways [40]. In experimental models IL-6 also induced the expression of plasminogen, which is a substrate for plasmin [41]. Plasmin-anti-plasmin levels were shown to be increased in patients with chronic inflammatory disorders such as systemic sclerosis [8], systemic lupus erythematosus, arthritis and other forms of collagen diseases [42]. The weak correlation between tPA and plasmin-anti-plasmin levels in our study suggests that activation of some tPA independent pathways contributed to elevation of plasmin-anti-plasmin complex. Accordingly some authors [43] underscored that not only tPA but also urokinase type plasminogen activator (u-PA) and factor XII dependent pathways may play a major role in plasminogen activation in inflammatory diseases.

 Endothelin-1 is a marker of endothelial dysfunction [43,44] and it has been shown to be involved in a spectrum of cardiovascular diseases ranging from coronary artery disease to PH. In our study ET-1 was increased in IPAH patients compared with controls and correlated positively with tPA and plasmin-anti-plasmin levels. This finding underscores endothelial cell activation in IPAH leading not only to the release of ET-1 but also plasminogen activators such as tPA or uPA, which further convert plasminogen to plasmin. 

 Of interest, in contrast to all other investigated markers, we found that the level of plasmin-anti-plasmin complex decreased after 3 months of PAH specific therapies. It was previously shown [45] that long-term prostacyclin infusion normalized platelet function and levels of endothelium derived clotting factors (factor VIII and von Willebrand factor). However, to the best of our knowledge, there are no published data showing the effects of PAH specific therapy on fibrinolytic markers. As plasmin-anti-plasmin complexes might reflect endothelial activation, it might be speculated that a reduction of their level reflects a beneficial effect of PAH specific therapy. The pathophysiological meaning and clinical consequence of this finding cannot be explained from our data and need further investigation. 

### Study limitations and strengths

 We chose only a limited number of haemostatic markers, namely tPA, plasmin-anti-plasmin, TAT, F1+2, PAI-1 and D-Dimers, from a broad variety of potential parameters. The chosen ones, however, represent critical steps in thrombogenesis and fibrinolysis, which was our focus of interest. To evaluate endothelial activation and inflammation we measured only two markers, ET-1 and IL-6, however both have a well defined role in the pathogenesis of PH [30,46]. This study allows no insight into causal relationships between parameters of the hemostasis, endothelial dysfunction and inflammation, which would require an experimental approach. 

 The interruption of VKA before catheterization might have been too short to fully abolish VKA effects on parameters of thrombogenesis. However, we did not observe significant differences of F1.2 and TAT between VKA-treated and VKA–naive IPAH patients.

 In this exploratory study we included a limited number of patients from one pulmonary hypertension centre. Therefore it is possible that some relevant associations could have been missed. Considering the low prevalence of IPAH in population a multicenter study involving large numbers of patients would be recommended to strengthen the validity of our results. 

 Strength of our study is the strictly defined IPAH population diagnosed according to current recommendations by international guidelines. Moreover, our data help in the search for the potential mechanisms underlying the recently observed increased bleeding risk in patients with IPAH.

## Conclusions

 In the present study we showed that markers of fibrinolysis were elevated in patients with IPAH however we did not find a clear evidence for increased thrombogenesis in this group of patients. Fibrinolysis, inflammation, and endothelial activation were closely interrelated in IPAH.

 In the light of the increased bleeding risk in IPAH further large scale studies on the role of the specific abnormalities of haemostasis in IPAH patients are warranted. 

## References

[1] Chaouat A, Weitzenblum E, Higenbottam T (1996) The role of thrombosis in severe pulmonary hypertension. Eur Respir J 9: 356-363. doi:10.1183/09031936.96.09020356. PubMed: 8777977. 8777977

[2] Stacher E, Graham BB, Hunt JM, Gandjeva A, Groshong SD et al. (2012) Modern age pathology of pulmonary arterial hypertension. Am J Respir Crit Care Med 186: 261-272. doi:10.1164/rccm.201201-0164OC. PubMed: 22679007. 22679007 PMC3886716

[3] Johnson SR, Mehta S, Granton JT (2006) Anticoagulation in pulmonary arterial hypertension: A qualitative systematic review. Eur Respir J 28: 999-1004. doi:10.1183/09031936.06.00015206. PubMed: 17074918. 17074918

[4] Galiè N, Hoeper MM, Humbert M, Torbicki A, Vachiery JL et al. (2009) Guidelines for the diagnosis and treatment of pulmonary hypertension: The task force for the diagnosis and treatment of pulmonary hypertension of the european society of cardiology (ESC) and the european respiratory society (ERS), endorsed by the international society of heart and lung transplantation (ISHLT). Eur Heart J 30: 2493-2537. doi:10.1093/eurheartj/ehp297. PubMed: 19713419. 19713419

[5] Johnson SR, Granton JT, Tomlinson GA, Grosbein HA, Le T et al. (2012) Warfarin in systemic sclerosis-associated and idiopathic pulmonary arterial hypertension. A bayesian approach to evaluating treatment for uncommon disease. J Rheumatol 39: 276-285. doi:10.3899/jrheum.110765. PubMed: 22247353. 22247353

[6] Peacock A (2013) Pulmonary hypertension. Eur Respir Rev 22: 20-25. doi:10.1183/09059180.00006912. PubMed: 23457160. 23457160 PMC9487435

[7] Henkens IR, Hazenoot T, Boonstra A, Huisman MV, Vonk-Noordegraaf A (2013) Major bleeding with vitamin K antagonist anticoagulants in pulmonary hypertension. Eur Respir J 41: 872-878. doi:10.1183/09031936.00039212. PubMed: 22936704. 22936704

[8] Jinnin M, Ihn H, Yamane K, Asano Y, Yazawa N et al. (2003) Plasma plasmin-alpha2-plasmin inhibitor complex levels are increased in systemic sclerosis patients with pulmonary hypertension. Rheumatology (Oxford) 42: 240-243. doi:10.1093/rheumatology/keg071. PubMed: 12595617. 12595617

[9] McLaughlin VV, Archer SL, Badesch DB, Barst RJ, Farber HW et al. (2009) ACCF/AHA 2009 expert consensus document on pulmonary hypertension a report of the american college of cardiology foundation task force on expert consensus documents and the american heart association developed in collaboration with the american college of chest physicians; american thoracic society, inc.; and the pulmonary hypertension association. J Am Coll Cardiol 53: 1573-1619. doi:10.1016/j.jacc.2009.01.004. PubMed: 19389575. 19389575

[10] Kozek E, Podolec P, Kopeć G, Pajak A, Tykarski A et al. (2008) Polish forum for prevention guidelines on diabetes. Kardiol Pol 66: 1020-1023. PubMed: 19004119. 19004119

[11] Kawecka-Jaszcz K, Jankowski P, Podolec P, Kopeć G, Naruszewicz M et al. (2008) Polish forum for prevention guidelines on smoking. Kardiol Pol 66: 125-126. doi:10.1140/epjb/e2008-00381-8. PubMed: 18363227. 18363227

[12] Cybulska B, Szostak WB, Podolec P, Kopeć G, Naruszewicz M et al. (2008) Polish forum for prevention guidelines on dyslipidaemia. Kardiol Pol 66: 1239-1242. PubMed: 19105106. 19105106

[13] Tykarski A, Podolec P, Kopeć G, Pajak A, Kawecka-Jaszcz K et al. (2007) Polish forum for prevention guidelines on arterial hypertension. Kardiol Pol 65: 1137-1141. PubMed: 18268817. 18268817

[14] Zahorska-Markiewicz B, Podolec P, Kopeć G, Drygas W, Godycki-Cwirko M et al. (2008) Polish forum for prevention guidelines on overweight and obesity. Kardiol Pol 66: 594-596. PubMed: 18630394. 18630394

[15] LaFarge CG, Miettinen OS (1970) The estimation of oxygen consumption. Cardiovasc Res 4: 23-30. doi:10.1093/cvr/4.1.23. PubMed: 5416840. 5416840

[16] Lippi G, Franchini M, Montagnana M, Salvagno GL, Poli G et al. (2006) Quality and reliability of routine coagulation testing: Can we trust that sample? Blood Coagul Fibrinolysis 17: 513-519. doi:10.1097/01.mbc.0000245290.57021.46. PubMed: 16988544. 16988544

[17] Eisenberg PR, Lucore C, Kaufman L, Sobel BE, Jaffe AS et al. (1990) Fibrinopeptide A levels indicative of pulmonary vascular thrombosis in patients with primary pulmonary hypertension. Circulation 82: 841-847. doi:10.1161/01.CIR.82.3.841. PubMed: 2394005. 2394005

[18] Welsh CH, Hassell KL, Badesch DB, Kressin DC, Marlar RA (1996) Coagulation and fibrinolytic profiles in patients with severe pulmonary hypertension. Chest 110: 710-717. doi:10.1378/chest.110.3.710. PubMed: 8797416. 8797416 10.1378/chest.110.3.710

[19] Hoeper MM, Sosada M, Fabel H (1998) Plasma coagulation profiles in patients with severe primary pulmonary hypertension. Eur Respir J 12: 1446-1449. doi:10.1183/09031936.98.12061446. PubMed: 9877507. 9877507

[20] Ling Y, Johnson MK, Kiely DG, Condliffe R, Elliot CA et al. (2012) Changing demographics, epidemiology, and survival of incident pulmonary arterial hypertension: Results from the pulmonary hypertension registry of the united kingdom and ireland. Am J Respir Crit Care Med 186: 790-796. doi:10.1164/rccm.201203-0383OC. PubMed: 22798320. 22798320

[21] Rich S, Dantzker DR, Ayres SM, Bergofsky EH, Brundage BH et al. (1987) Primary pulmonary hypertension. A national prospective study. Ann Intern Med 107: 216-223. doi:10.7326/0003-4819-107-2-216. PubMed: 3605900. 3605900

[22] Christ G, Graf S, Huber-Beckmann R, Zorn G, Lang I et al. (2001) Impairment of the plasmin activation system in primary pulmonary hypertension: Evidence for gender differences. Thromb Haemost 86: 557-562. PubMed: 11522003. 11522003

[23] Huber K, Beckmann R, Frank H, Kneussl M, Mlczoch J et al. (1994) Fibrinogen, t-PA, and PAI-1 plasma levels in patients with pulmonary hypertension. Am J Respir Crit Care Med 150: 929-933. doi:10.1164/ajrccm.150.4.7921465. PubMed: 7921465. 7921465

[24] Miniati M, Fiorillo C, Becatti M, Monti S, Bottai M et al. (2010) Fibrin resistance to lysis in patients with pulmonary hypertension other than thromboembolic. Am J Respir Crit Care Med 181: 992-996. doi:10.1164/rccm.200907-1135OC. PubMed: 20075386. 20075386

[25] Cesarman-Maus G, Hajjar KA (2005) Molecular mechanisms of fibrinolysis. Br J Haematol 129: 307-321. doi:10.1111/j.1365-2141.2005.05444.x. PubMed: 15842654. 15842654

[26] Emeis JJ (1992) Regulation of the acute release of tissue-type plasminogen activator from the endothelium by coagulation activation products. Ann N Y Acad Sci 667: 249-258. doi:10.1111/j.1749-6632.1992.tb51622.x. PubMed: 1309043. 1309043

[27] van Hinsbergh VW (2012) Endothelium--role in regulation of coagulation and inflammation. Semin Immunopathol 34: 93-106. doi:10.1007/s00281-011-0285-5. PubMed: 21845431. 21845431 PMC3233666

[28] Levi M, van der Poll T, Büller HR (2004) Bidirectional relation between inflammation and coagulation. Circulation 109: 2698-2704. doi:10.1161/01.CIR.0000131660.51520.9A. PubMed: 15184294. 15184294

[29] Budhiraja R, Tuder RM, Hassoun PM (2004) Endothelial dysfunction in pulmonary hypertension. Circulation 109: 159-165. doi:10.1161/01.CIR.0000102381.57477.50. PubMed: 14734504. 14734504

[30] Price LC, Wort SJ, Perros F, Dorfmuller P, Huertas A et al. (2012) Inflammation in pulmonary arterial hypertension. Chest 141: 210-221. doi:10.1378/chest.11-0793. PubMed: 22215829. 22215829

[31] Soon E, Holmes AM, Treacy CM, Doughty NJ, Southgate L et al. (2010) Elevated levels of inflammatory cytokines predict survival in idiopathic and familial pulmonary arterial hypertension. Circulation 122: 920-927. doi:10.1161/CIRCULATIONAHA.109.933762. PubMed: 20713898. 20713898

[32] Hiremath J, Thanikachalam S, Parikh K, Shanmugasundaram S, Bangera S et al. (2010) Exercise improvement and plasma biomarker changes with intravenous treprostinil therapy for pulmonary arterial hypertension: A placebo-controlled trial. J Heart Lung Transplant 29: 137-149. doi:10.1016/j.healun.2009.09.005. PubMed: 20022264. 20022264

[33] Savale L, Tu L, Rideau D, Izziki M, Maitre B et al. (2009) Impact of interleukin-6 on hypoxia-induced pulmonary hypertension and lung inflammation in mice. Respir Res 10: 6-9921-10-6 PubMed: 19173740. 19173740 10.1186/1465-9921-10-6 PMC2644669

[34] Damås JK, Otterdal K, Yndestad A, Aass H, Solum NO et al. (2004) Soluble CD40 ligand in pulmonary arterial hypertension: Possible pathogenic role of the interaction between platelets and endothelial cells. Circulation 110: 999-1005. doi:10.1161/01.CIR.0000139859.68513.FC. PubMed: 15302794. 15302794

[35] Kruithof EK, Mestries JC, Gascon MP, Ythier A (1997) The coagulation and fibrinolytic responses of baboons after in vivo thrombin generation--effect of interleukin 6. Thromb Haemost 77: 905-910. PubMed: 9184401. 9184401

[36] Wannamethee SG, Sattar N, Rumley A, Whincup PH, Lennon L et al. (2008) Tissue plasminogen activator, von willebrand factor, and risk of type 2 diabetes in older men. Diabetes Care 31: 995-1000. doi:10.2337/dc07-1569. PubMed: 18235054. 18235054

[37] Yudkin JS, Stehouwer CD, Emeis JJ, Coppack SW (1999) C-reactive protein in healthy subjects: Associations with obesity, insulin resistance, and endothelial dysfunction: A potential role for cytokines originating from adipose tissue? Arterioscler Thromb Vasc Biol 19: 972-978. doi:10.1161/01.ATV.19.4.972. PubMed: 10195925. 10195925

[38] Wannamethee SG, Whincup PH, Rumley A, Lowe GD (2007) Inter-relationships of interleukin-6, cardiovascular risk factors and the metabolic syndrome among older men. J Thromb Haemost 5: 1637-1643. doi:10.1111/j.1538-7836.2007.02643.x. PubMed: 17596140. 17596140

[39] Páramo JA, Sangro B, Prósper F, Quiroga J, Rifón J et al. (1995) Increased concentrations of tumor necrosis factor and interleukin-6 contribute to the hemostatic abnormalities in advanced liver disease. Haemostasis 25: 305-311. PubMed: 8586322. 8586322 10.1159/000217177

[40] Kerr R, Stirling D, Ludlam CA (2001) Interleukin 6 and haemostasis. Br J Haematol 115: 3-12. doi:10.1046/j.1365-2141.2001.03061.x. PubMed: 11722403. 11722403

[41] Jenkins GR, Seiffert D, Parmer RJ, Miles LA (1997) Regulation of plasminogen gene expression by interleukin-6. Blood 89: 2394-2403. PubMed: 9116283. 9116283

[42] Kawakami M, Kawagoe M, Harigai M, Hara M, Hirose T et al. (1989) Elevated plasma levels of alpha 2-plasmin inhibitor-plasmin complex in patients with rheumatic diseases. possible role of fibrinolytic mechanism in vasculitis. Arthritis Rheum 32: 1427-1433. doi:10.1002/anr.1780321112. PubMed: 2530990. 2530990

[43] Paulus P, Jennewein C, Zacharowski K (2011) Biomarkers of endothelial dysfunction: Can they help us deciphering systemic inflammation and sepsis? Biomarkers 16 Suppl 1: S11-S21. doi:10.3109/1354750X.2011.587893. PubMed: 21707440. 21707440

[44] Abraham D, Dashwood M. (2008) Endothelin--role in vascular disease. Rheumatology (Oxford) 47 Suppl 5: v23-4. 10.1093/rheumatology/ken282 18784133

[45] Friedman R, Mears JG, Barst RJ (1997) Continuous infusion of prostacyclin normalizes plasma markers of endothelial cell injury and platelet aggregation in primary pulmonary hypertension. Circulation 96: 2782-2784. doi:10.1161/01.CIR.96.9.2782. PubMed: 9386137. 9386137

[46] Davie NJ, Schermuly RT, Weissmann N, Grimminger F, Ghofrani HA (2009) The science of endothelin-1 and endothelin receptor antagonists in the management of pulmonary arterial hypertension: Current understanding and future studies. Eur J Clin Invest 39 Suppl 2: 38-49. doi:10.1111/j.1365-2362.2009.02120.x. PubMed: 19335746. 19335746

